# Effects of Total Parenteral Nutrition on Serum Osmolality and Patent Ductus Arteriosus

**DOI:** 10.7759/cureus.64196

**Published:** 2024-07-09

**Authors:** Yukiko Toya, Kotaro Oyama, Shigekuni Tsuchiya, Atsushi Matsumoto, Nao Takashimizu, Fumiaki Takahashi, Manami Akasaka

**Affiliations:** 1 Department of Pediatrics, School of Medicine, Iwate Medical University, Shiwa, JPN; 2 Department of Information Science, School of Medicine, Iwate Medical University, Shiwa, JPN

**Keywords:** early aggressive nutrition, patent ductus arteriosus, serum osmolality, very preterm infants, total parenteral nutrition

## Abstract

Background: The persistence of high serum osmolality in the early postnatal period is a risk for developing patent ductus arteriosus (PDA). Early aggressive nutrition (EAN), involving total parenteral nutrition (TPN), by which enough concentrations of glucose and amino acids are administered intravenously, is recommended postnatally to improve the neurological prognosis in preterm infants. However, the effects of EAN involving TPN on serum osmolality and the development of a PDA have not been adequately studied.

Objectives: Thus, in this study, we aimed to investigate the impact of TPN on serum osmolality and determine whether increased serum osmolality could be associated with a higher incidence of PDA in preterm infants.

Methods: In this single-center retrospective observational study, preterm infants born at <28 weeks of gestation who had been admitted to our neonatal intensive care unit (NICU) before (pre-TPN period) and after the introduction of TPN (post-TPN) were included. We reviewed the medical records of these patients, compared the changes in serum osmolality from birth to five days after birth, the clinical background, and the incidence of PDA between these two periods, and analyzed the risk factors. Additionally, the factors affecting the serum osmolality in very preterm infants were examined. The patients who met the intervention criteria of our NICU and received a cyclooxygenase (COX) inhibitor, Indacin^®^ (Nobelpharma, Tokyo, Japan), within seven days after birth were classified as PDA+; those who could not be identified to have PDA flow by echo and did not receive a COX inhibitor were classified as PDA-.

Results: The postnatal day and serum sodium (Na^+^) were statistically significantly correlated with a higher serum osmolality. Serum osmolality remained statistically significantly higher in the PDA+ cohort compared with the PDA- cohort after the first day of life. However, no statistically significant differences were observed in serum osmolality after 24 hours of age, weeks of gestational age, birth weight, or incidence of PDA between the pre- and post-TPN periods. The results of the multiple logistic regression analyses revealed that the increased serum osmolality correlated with PDA development.

Conclusions: In this study, the serum Na^+^ statistically significantly correlated with a higher serum osmolality. Moreover, the increased serum osmolality correlated with PDA development. Thus, the prevention of hypernatremia might reduce the incidence of PDA. Nonetheless, the findings in this study revealed that no statistically significant differences in serum osmolality were observed between the pre-and post-TPN periods, indicating that TPN had little effect on serum osmolality.

## Introduction

Preterm patent ductus arteriosus (PDA) is caused by a lack of development in the structure and function of the ductus arteriosus (DA). Various mechanisms underlying the closure of the DA include endometrial remodeling, beginning in the fetal period [1] to around the postnatal spontaneous closure of the DA [2]; increased postnatal partial pressure of the arterial blood oxygen [3-4]; decreased serum prostaglandin concentration [3-4]; and recently, the involvement of serum osmolality [5-7]. Furthermore, Aoki et al. [6] demonstrated that serum osmolality is not adequately reduced at the time of birth in very preterm infants at <28 weeks of gestation, who are more likely to develop PDA. Conversely, serum osmolality is reduced at the time of birth in preterm infants born at ≥28 weeks of gestation, comparable to that of full-term infants [6].

During the early postnatal period, inadequate nutrition in very-low-birth-weight infants (VLBWIs) potentially results in cerebral palsy and cognitive decline [8]. Total parenteral nutrition (TPN) is a transvenous nutritional management method that involves administering sufficient glucose, amino acids, and fat products to the VLBWIs before the establishment of enteral nutrition [9,10]. Due to the fragility of the intestinal tract and the need for close fluid management in VLBWIs, providing adequate enteral nutrition in the early postnatal period is not feasible. Therefore, the importance of early aggressive nutrition (EAN), with the immediate postnatal initiation of TPN, has been advocated [10]. However, in preterm infants who have immature renal function, concerns remain regarding metabolic acidosis and hyperammonemia due to protein loading during EAN. In addition, amino acids increase insulin secretion, which requires administering high concentrations of glucose solutions to maintain the blood glucose and caloric nitrogen ratio. Therefore, TPN can potentially increase blood urea nitrogen (BUN), glucose levels, and serum osmolality.

Nonetheless, since the effects of EAN on the serum osmolality and development of a PDA have not been adequately established, our neonatal intensive care unit (NICU) policy is to initiate TPN after the closure of PDA. Consequently, in this study, we aimed to assess the changes in serum osmolality in preterm infants before and after the introduction of TPN, evaluate the relationship between serum osmolality and the incidence of PDA in preterm infants, and identify specific factors, including serum sodium (Na+) levels, that could influence serum osmolality in preterm infants receiving TPN. Logistic regression and repeated-measures analysis of variance were used to retrospectively analyze data collected from preterm infants and construct receiver operating characteristic (ROC) curves to assess the predictive power of significant factors.

## Materials and methods

Ethical considerations

This study was approved by the ethics committee (EC) of Iwate Medical University, Shiwa, Japan (EC approval number: H28-198). Consent to participate in the study was obtained from family members via opt-out. This study was conducted following the principles of the Declaration of Helsinki.

Study design

This was a retrospective cohort study. The inclusion criteria were preterm infants born at less than 28 weeks of gestation and admitted to the NICU within 24 hours after birth. The exclusion criteria included congenital anomalies, early death (within 48 hours), or incomplete medical records. The pre-TPN period encompassed years from 2006 to 2008, while the post-TPN period encompassed years from 2012 to 2014. The period of 2009-2011 was excluded owing to the unavailability of amino acid preparations for neonates in the hospital and the fact that the type of amino acid preparations varied from case to case.

Two independent researchers extracted data from electronic medical records using a standardized form. Discrepancies were resolved by reaching a consensus. Serum osmolality was measured daily for the first three days of life. Patent ductus arteriosus was diagnosed based on echocardiographic criteria, including a ductal diameter >1.5 mm.

Total parenteral nutrition dosing method

At our hospital, TPN has been indicated for VLBWIs that are <1500 g. With a pending closure of the DA, TPN was initiated with a glucose concentration of 4-5 g/kg/day, amino acids of 0.5-1 g/kg/day, and was increased by 0.5 g/kg/day every 24 h until reaching a target dose.

A comprehensive amino acid preparation for pediatric TPN, Preamine P® (Fuso Pharmaceutical Co., Ltd., Osaka, Japan), was used. Additionally, the glucose concentration was adjusted using serum glucose as an index while targeting a carbon-to-nitrogen ratio of 200:300. An intravenous infusion of fat emulsion comprised a purified soybean oil, 20% Intralipos® (Otsuka Pharmaceutical Co., Ltd., Tokyo, Japan), initiated at 0.25-0.5 g/kg with a maximum dose of 1 g/kg. Total parenteral nutrition was administered until the total enteral feeding exceeded 100 mL/kg/day. Furthermore, care was taken to avoid metabolic acidosis caused by the amino acid preparations and hyperammonemia, as well as the worsening of the respiratory status due to the intravenous infusion of fat emulsion.

Intervention criteria for PDA

In our NICU, we evaluate the DA using echocardiography every six to 12 hours after birth. This is the protocol we have established in our NICU. Echocardiographic findings included a DA diameter >1.4 mm [11, 12], a left atrium/aortic ratio (LA/Ao) of >1.5 [11, 12], and a PDA flow pattern of the growing or pulsatile type [13]. Patients with at least one of these echocardiographic findings and at least three points on the cardiovascular dysfunction score were included [14]. The cardiovascular dysfunction score comprised parameters including the pulse rate, heart murmur, cardiothoracic ratio, and precordial pulsation. In addition, the patients who did not meet any of these criteria but were expected to develop PDA due to the worsening of echocardiographic findings were included.

Cohorts of PDA+ and PDA-

Patients who met the intervention criteria and received a cyclooxygenase (COX) inhibitor, Indacin® (Nobelpharma, Tokyo, Japan), within seven days after birth were classified as PDA+, and those who did not receive a COX inhibitor were classified as PDA-.

The patients in the PDA+ cohort were treated with this COX inhibitor at 0.05-0.1 mg/kg every 12-24 hours for a course comprising three doses until ductal closure. The maximum number of courses was two, with six doses in total. If ductal closure was confirmed during the middle of the course, the administration of the COX inhibitor was discontinued.

Maternal and neonatal factors

We retrospectively reviewed the medical records of the infants, comparing the changes in serum osmolality, clinical background, and incidence of PDA between the pre-and post-TPN periods. We compared the maternal and neonatal factors in the PDA+ and PDA- cohorts and analyzed these risk factors in the development of PDA using multiple logistic regression analysis.

As presented in Table 1, eight maternal factors were extracted, including age, pregnancy complications, and drug administration. Furthermore, 36 neonatal factors were extracted, including perinatal information, complications, vital signs up to three days postnatally, blood chemistry, drug administration, and echocardiographic findings.

**Table 1:** Clinical background of the subject infants Median (interquartile range) In the post-TPN period, the number of boys was higher, and their Apgar score at one minute was lower. Serum osmolality was significantly higher in the post-TPN period only after birth, but not significantly different thereafter. TPN: total parenteral nutrition; PDA: patent ductus arteriosus; OSM: serum osmolality

	Pre-TPN period	Post-TPN period	P-value
n	83	74	
Gestational age (weeks)	25.7 (2.4)	25.4 (2.6)	0.515
Birth weight (g)	756 (263)	749 (287.3)	0.52
Sex (male, %)	45.8	64.9	0.017
PDA (%)	57.8	73	0.093
Onset time of PDA (h)	26	24	0.13
Apgar score at 1 min	4 (2.5)	3 (2)	0.018
Apgar score at 5 min	7 (2)	6 (1)	0.062
Steroid administration after birth (%)	4.8	21.9	0.001
Clinical CAM (%)	44.6	49.3	0.611
OSM (day 0)	282.6 (8.8)	285.0 (7.6)	0.025
OSM (day 1)	296.2 (14.3)	300.8 (9.3)	0.153
OSM (day 2)	310.0 (12.4)	311.5 (12.7)	0.488
OSM (day 3)	306.6 (14.8)	311.1 (18.4)	0.281

Serum osmolality was measured daily for the first three days of life. Serum osmolality was calculated using the formula as follows:

Serum osmolality = 2 (Na^+^ +K^+^) + blood glucose/18 + BUN/2.8 (1)

where Na^+^ and K^+^ represent sodium and potassium, respectively [6].

Congenital infection was defined as an immunoglobulin M >20 mg/dL at birth or cases of culture-positive specimens obtained from sterile sites.

Statistical analyses

All quantitative data were expressed as medians and interquartile ranges unless otherwise indicated. Missing data were handled using multiple imputation techniques. Sensitivity analyses were performed to assess the impact of missing data on the results. The Mann-Whitney U test was used to compare median weights between the groups. Logistic regression included the variables gestational age, fraction of inspired oxygen (FIO_2_), and osmolality immediately after birth and day one, which were identified as significant using univariate analysis. The ROC curves were constructed using IBM SPSS Statistics software for Windows, version 23.0 (IBM Corp., Armonk, NY), with the area under the curve (AUC) values calculated to assess predictive accuracy. A general linear model for repeated measurements was performed regarding the post-TPN period. The effect of the covariates was examined by fitting a model in which the outcome variable was serum osmolality. The covariates included age in days, serum Na^+^, and the presence or absence of TPN. The within-subject correlations were modeled using an unstructured covariance matrix. Missing data were handled using multiple imputation techniques. Sensitivity analyses were performed to assess the impact of missing data on the results.

IBM SPSS statistical software for Windows was used for the statistical analyses. Two-tailed statistical significance was set at p < 0.05. Statistical power analysis was performed using G* Power statistical software, version 3.1.9.2 (Heinrich-Heine-Universität Düsseldorf, Düsseldorf, Germany). When α = 0.05 and the power was set to 0.8 under the condition that the effect size was guaranteed to be 0.5, the number of patients was calculated to be 64 and 64 for the pre-and post-TPN periods, respectively.

## Results

**Figure 1:**
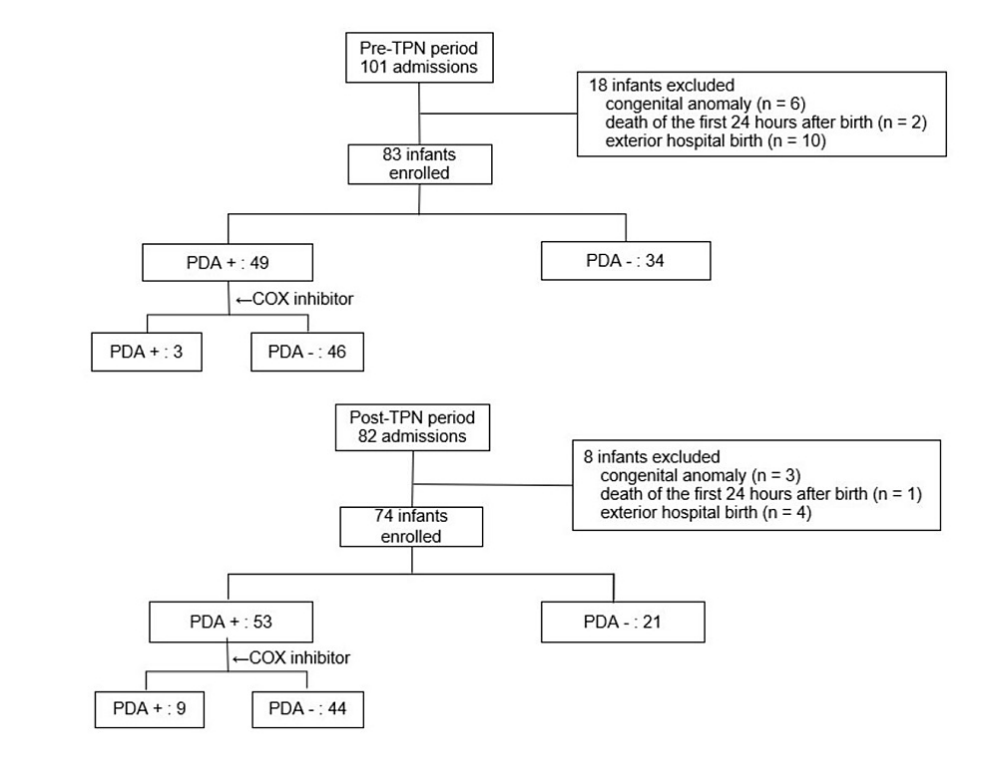
Flowchart showcasing hospitalization data during the two target periods In the pre-TPN period, 83 patients were included, of whom 18 were excluded. Similarly, 74 patients were included in the post-TPN period, of whom eight were excluded. In the former period, 49 patients developed PDA, and in the latter period, 53 developed PDA and were treated with COX inhibitors. TPN: total parenteral nutrition; PDA: patent ductus arteriosus; COX: cyclooxygenase

Eighty-three and 74 infants were admitted to the NICU during the pre-and post-TPN periods, respectively (Figure 1).

The median weeks of pregnancy and birth weight for the pre-TPN period were 25.7 weeks and 756 g, respectively, while those for the post-TPN period were 25.4 weeks and 749 g, respectively. No statistically significant differences were observed between the two periods (p = 0.515 and p = 0.520, respectively).

Moreover, statistically significant differences were not observed in the incidence or time of the onset of the PDA between the two periods (p = 0.093, p = 0.13, respectively). The number of male patients was statistically significantly higher; Apgar scores at one minute were statistically significantly lower (p = 0.017, p = 0.018, respectively) in the post-TPN period than in the pre-TPN period.

Serum osmolality was statistically significantly elevated at two to three days after birth (p< 0.000). Serum osmolality was statistically significantly higher in the post-TPN period immediately postnatally than in the pre-TPN period postnatally (p = 0.025). However, no statistically significant differences were observed after the first day of life. The infants in the post-TPN period were statistically significantly more likely to receive postnatal steroids compared with those in the pre-TPN period (p = 0.001) (Table 1).

In the post-TPN period, 0%, 21.6%, 58.1%, and 81.1% of the infants received TPN on the day of birth and postnatal days one, two, and three, respectively. In the general linear model for repeated measurements, the postnatal day and serum Na^+^ statistically significantly correlated with a higher serum osmolality (p<0.001, p<0.001, respectively). Furthermore, the correlation between serum osmolality and the presence of TPN was not statistically significant (p = 0.582) (Table 2).

**Table 2:** Statistical analysis of the factors affecting serum osmolality The postnatal day and serum Na^+ ^were statistically significantly correlated with a higher serum osmolality and the correlation between the serum osmolality and the presence of TPN was not statistically significant. TPN: total parenteral nutrition; SE: standard error; Pr: probability

		Estimate	SE	t value	Pr > |t|
Intercept		34.7659	9.7637	3.56	0.0007
TPN-		Reference			
TPN+		-0.5671	0.8948	-0.63	0.5282
Na		1.9275	0.06916	27.87	< .0001>
Day after birth		2.9171	0.4765	6.12	< .0001>
(Day after birth)^2^		-2.7065	0.2587	-10.46	<0.001

A comparative analysis revealed no statistically significant differences in the maternal factors or days of hospitalization between the PDA+ and PDA- cohorts. However, the weeks of gestational age were less in the PDA+ cohort (p = 0.043) than in the PDA- cohort. No statistically significant differences were found in the birth weight and Apgar scores between the two cohorts. The FIO_2_ immediately postnatally and serum osmolality on postnatal days one to three were statistically significantly higher in the PDA+ cohort than in the PDA- cohort (p = 0.004, p = 0.003, p = 0.003, and p = 0.003, respectively) (Table 3).

**Table 3:** Comparison of clinical data between PDA+ and PDA- groups Median (interquartile range) PDA+ had significantly shorter gestational weeks and required more oxygen immediately after birth. Serum osmolality was significantly higher in PDA+ at one to three days after birth. PDA: patent ductus arteriosus; PDA+: patients who met the intervention criteria of our neonatal intensive care unit and received a cyclooxygenase (COX) inhibitor, Indacin® within seven days after birth were classified as PDA+; PDA-: those who could not be identified to have PDA flow by echo and did not receive a COX inhibitor were classified as PDA-; FiO_2_: fraction of inspired oxygen; OSM: serum osmolality; GIR: glucose infusion ratio

	PDA+	PDA-	P-value
Gestational age (weeks)	25.4 (2.6)	26.4 (2)	0.043
Birth weight (g)	749 (286)	762 (307)	0.851
Period B (%)	51	39	0.093
Apgar score at 1 minute	3 (3)	4 (3)	0.398
Apgar score at 5 min	6 (1)	7 (2)	0.062
FiO_2_ (day 0)	0.98 (0.4)	0.6 (0.6)	0.004
FiO_2_ (day 1)	0.24 (0.04)	0.24 (0.05)	0.731
FiO_2_ (day 2)	0.25 (0.05)	0.25 (0.05)	0.387
FiO_2_ (day 3)	0.24 (0.02)	0.23 (0.05)	0.755
OSM (day 0)	285.0 (10.0)	282.6 (10.9)	0.060
OSM (day 1)	301.5 (19.3)	295.2 (17.0)	0.003
OSM (day 2)	313.2 (15.1)	307.0 (18.7)	0.003
OSM (day 3)	311.4 (19.6)	300.4 (25.5)	0.003
GIR (day 0) (mg/kg/min)	2.9 (0.7)	2.95 (0.6)	0.625
GIR (day 1) (mg/kg/min)	4.2 (2.0)	3.8 (1.2)	0.322
GIR (day 2) (mg/kg/min)	5.7 (2.6)	5 (2.5)	0.010
GIR (day3) (mg/kg/min)	5.7 (3.3)	5.6 (2.2)	0.209

The LA/Ao on postnatal day one was higher in the PDA+ cohort than in the PDA- cohort (p = 0.000). Furthermore, the multiple logistic regression analysis with the PDA onset as the dependent variable revealed a statistically significant correlation between the FIO_2_ and serum osmolality on postnatal day one (p = 0.024, 95% confidence interval 1.221-16.271; p = 0.012, 95% confidence interval 1.007-1.060, respectively). The AUC values were 0.644 and 0.633 for the PDA+ and PDA- cohorts, respectively.

## Discussion

Summary and significance of the main findings

This study analyzed the factors that affected serum osmolality, focusing on serum Na^+^ levels. The findings in this study revealed that no statistically significant differences in serum osmolality were found between the pre-and post-TPN periods, indicating that TPN had little effect on serum osmolality.

Comparison with the findings of previous studies

One of the major strengths of this study is its focus on a vulnerable population of preterm infants born at <28 weeks of gestation, providing valuable insights into their nutritional management. The use of multiple logistic regression and ROC curves could allow for a robust analysis of risk factors and predictive accuracy. The study contributes to the limited literature on the relationship between TPN, serum osmolality, and PDA, highlighting important clinical considerations.

Serum osmolality is more likely to increase with concomitant hypernatremia and hyperglycemia than with hyperglycemia alone [15]. A study by Gawlowski et al. revealed that 69.7% of preterm infants born at <27 weeks of gestational age are predisposed to hypernatremia, peaking at 24-48 hours of postnatal age. Furthermore, this population was found to be more likely to develop chronic lung disease, a PDA, or intracerebral hemorrhage [16]. In this previous study, no differences in birth weight or sex were found; the rate of water loss improved with fluid loading, suggesting a high rate of water loss due to premature birth.

Skin keratinization begins around 18 weeks of gestation but remains insufficient at 26 weeks [17-18]. Percutaneous water loss is 20-40 mL/kg in full-term infants and 200 mL/kg at ≤ 26 weeks of gestation [19].

Additionally, polyuria in early life and the inability of immature kidneys to respond to changes in fluid delivery and electrolytes contribute to increased serum osmolality [15]. Therefore, hypernatremia and elevated serum osmolality are risks at birth in very preterm infants. Total parenteral nutrition has little effect on serum osmolality, and EAN should be promoted to improve the neurological prognosis of preterm infants.

Serum osmolality remained statistically significantly higher in the PDA+ cohort compared with the PDA- cohort after the first day of life. Furthermore, the results of the multiple logistic regression analyses revealed a correlation between increased serum osmolality and the development of a PDA, consistent with the findings of previous studies. The findings of a study conducted in rats by Aoki et al. revealed that a decrease in serum osmolality was correlated with ductal closure via the hypo-osmotic sensor transient receptor potential melastatin 3 (TRPM3). TRPM3 is abundant in the intima of the DA and senses a decrease in serum osmolality, thus promoting ductal closure. However, this response does not occur in the aorta. In humans, preterm infants born at 28-35 weeks of gestation exhibit a decrease in serum osmolality similar to that of normal-term infants immediately postnatally, followed by a gradual recovery. Conversely, very preterm infants born at 24-27 weeks of gestation do not exhibit a decrease in serum osmolality; nevertheless, a rapid rise within two to three days postnatally is observed [6]. This trend was supported and more pronounced in our study in the PDA+ cohort.

Among the various risk factors, we found that the number of weeks of gestational age strongly correlates with the development of PDA. At <28 weeks of gestational age, birth itself is a risk factor for the development of PDA. Thus, we consider that adequate fluid loading and the prevention of transepidermal water loss will avert hypernatremia and increase serum osmolality, which are the underlying causes of prematurity, thus decreasing the incidence of a PDA.

Study limitations

Although our study provides important insights into the relationship between TPN and PDA in preterm infants, several limitations must be acknowledged. A notable limitation of this study is its retrospective design, which might have limited the ability to establish causation and introduced potential biases related to data extraction from the medical records. The incremental increase in TPN doses might not have accurately reflected the effects of higher initial doses on serum osmolality and PDA development. Future studies should consider standardized TPN administration protocols. The absence of data on immediate postnatal TPN initiation means that we cannot evaluate the potential benefits or risks associated with EAN protocols. Prospective studies are needed to address this gap. The single-center study design might limit the generalizability of our findings to other NICUs with different patient populations and clinical practices. Despite these limitations, the study’s strengths include the focus on a highly vulnerable population, the use of robust statistical methods to analyze risk factors, and predictive accuracy. Given the retrospective design and the incremental increase in TPN doses, our findings might not fully capture the effects of immediate, high-dose TPN administration on serum osmolality and PDA development. Prospective studies with standardized protocols are needed to confirm these results.

## Conclusions

Our results indicated a significant correlation between serum Na^+^ and osmolality, suggesting that careful monitoring and management of Na^+^ levels in preterm infants receiving TPN could potentially reduce the risk of PDA. Clinicians should consider strategies for preventing hypernatremia, such as adjusting electrolyte composition in TPN solutions.

Although TPN appears to have little effect on serum osmolality overall, our findings highlight the importance of individualized nutrition plans. By closely monitoring serum osmolality and Na^+^ levels and adjusting TPN composition accordingly, healthcare providers could mitigate the risk factors associated with PDA in preterm infants.
